# Giant splenic pseudocyst, a rare aetiology of abdominal tumor: a case report

**DOI:** 10.1186/1757-1626-3-16

**Published:** 2010-01-11

**Authors:** Mamadou Cissé, Ibrahima Konaté, Ousmane Ka, Madieng Dieng, Abdarahmane Dia, Cheikh T Touré

**Affiliations:** 1 Clinique Chirurgicale, Hpital Aristide Le Dantec, Avenue Pasteur, Dakar, BP 3001, Sénégal

## Abstract

**Introduction:**

Splenic pseudocysts are nonparasitic cyst without epithelial lining. We report this case especially by its way of revelation, its large size and its per operative presentation which needed total splenectomy. To this opportunity, we discuss the diagnostic procedure and therapeutic indications.

**Case presentation:**

A twenty-year old Senegalese woman, was admitted with a three-month history of spontaneous abdominal mass associated with a pain. Ultrasonography and CT scan found the giant splenic pseudocyst with a diameter of 20 cm which needed a total splenectomy by median laparotomy.

**Conclusion:**

Usually, symptomless splenic cysts are untreated. When surgical treatment is indicated, recommendations are to preserve splenic parenchyma by partial splenectomy or fenestration especially by laparoscopy. Total splenectomy retains some guidance.

## Background

Splenic pseudocysts are nonparasitic cyst without epithelial lining [1]. Those are mostly secondary to post traumatic haematomas. Without a recent trauma history, there is no clinical or radiological feature which distinguishes them to epidermoid cyst [2]. Distinction may be made at histology. Surgical management of these symptomatic pseudocysts promotes spleen parenchyma preservation. We report this case especially by its way of revelation, its large size which needed total splenectomy. To this opportunity, we discuss the diagnostic procedure and therapeutic indications.

## Case presentation

**Figure 1:**
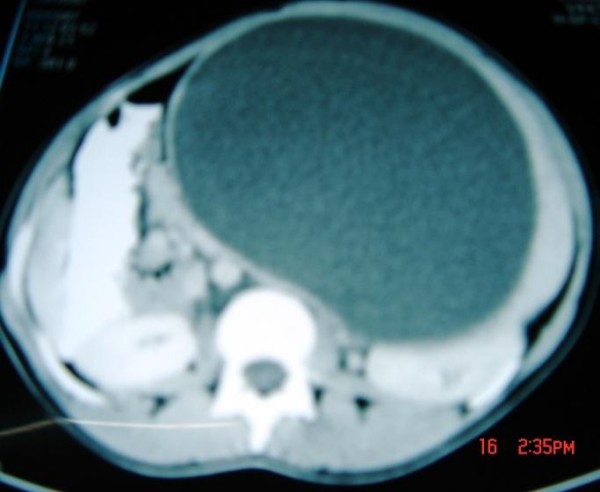
**Abdominal CT scan showing a giant splenic pseudocyst with net lining, and a diameter of 20 cm**.

**Figure 2:**
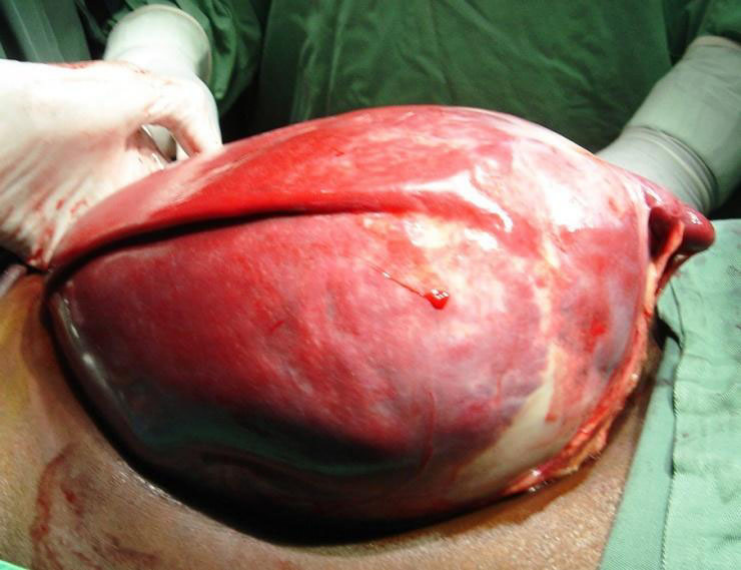
**Per operative view of pseudotumor cystic spleen with a rich vascular area**.

A twenty-year-old Senegalese black woman, was admitted with a three-months history of spontaneous abdominal mass associated with a pain. There were no histories of trauma or infection. On examination, there were an abdominal mass in the left hypochondrium and epigastric area. This mass was steady, with a diameter of 20 cm. Hydatid serology was negative and blood formula was normal. Ultrasonography found a voluminous mixed fluid mass without specifying its origin. CT scan found a splenic cyst mass with net lining, regular, without calcifications, without enhancement after contrast injection (Figure 1). Its content was homogeneous liquid without tissue structure. She referred to a splenic cyst with splenic parenchyma remaining below 25%. A median laparotomy found a splenic pseudotumor cyst with a rich vascular area (Figure 2) and the persistence of substantial thickness splenic parenchyma encompassing cyst. A total splenectomy was performed. The postoperative care consisted of a vaccination against meningococcus, pneumococcus, and Haemophilus influenzae and antibiotic-based Oracilline. Her progress has been satisfactory. The pathological examination of surgical specimen found a pseudo cyst from resorption of splenic hematoma without an epithelial lining, with a weight of 4100 g.

## Discussion

Management of these splenic cyst is threefold: What is the nature of the cyst? Should we operate and what surgery do? Cysts are classified as congenital cyst, pseudocyst, parasitic and tumor cysts. Our case was nonparasitic splenic cysts in the absence of infectious syndrome and non-tumoral on imaging. Martin Fowler [1], have made a classification distinguishing between congenital cysts (CC) that have an epithelial lining and post traumatic pseudo cysts (PC) which do not. Therefore, the distinction between CC and pseudo cyst may be made at histology. Without the recent notion obvious trauma, there is no clinical or radiological feature which distinguish them. They appear both in the CT scan thin-walled, hypodense, and without enhancement after contrast injection [2]. This distinction based on the presence or absence of epithelial lining is challenged by Morgenstern [3], which considers that several CC are wrongly considered as PC. Indeed, the wall is discontinuous and only a careful histological reading layout to suggest his absence or his presence. The lack of trauma and epithelial lining induced our case was a pseudo cyst by resorption of a hematoma. The second concern relates to the operative indication. Usually, symptomless splenic cysts are untreated. Indeed, the complications over time are rare. Cowles [4], found, in a total of 191 cases monitored a complication rate of 5.2% in type of rupture and infection. Our surgical indication was based on the size of splenic pseudocyst which carried out an abdominal mass exposed to a risk of rupture. The surgical treatment of these splenic cysts is partial or total splenectomy, and fenestration. Laparoscopic fenestration, however, is associated with a significant recurrence rate. Schier [5], found a recurrence rate of 64% within an average of 1 year, despite all the procedures used to treat residual cavity (omentum package, argon destruction). To prevent the complications of total splenectomy, partial splenectomy appears to be the best indication but leaving at least 25% of the splenic parenchyma and with access to effective means of sliced section hemostasis. We conducted a total splenectomy because of an insufficient residual parenchyma and the high risk of bleeding from a partial splenectomy.

## Conclusion

Splenic pseudo cysts are benign formations often secondary to the resorption of a hematoma. Asymptomatic forms do not belong to any treatment. When they are symptomatic, partial splenectomy is superior to laparoscopic fenestration in terms of recurrence. Total splenectomy retains some guidance.

## Consent

Written informed consent was obtained from the patient for publication of this case report and accompanying images. A copy of the written consent is available for review by the Editor-in-Chief of this journal.

## Competing interests

The authors declare that they have no competing interests.

## Authors' contributions

MC performed the surgical procedure and reported the case. IK and OK interpreted and analysed the tomodensitometry findings. MD participate to diagnostic and therapeutic decisions. AD and CT have major contribution in writing the manuscript. All authors read and approved the final manuscript.
